# Integrating weighted gene co-expression network analysis and machine learning to elucidate neural characteristics in a mouse model of depression

**DOI:** 10.3389/fpsyt.2025.1564095

**Published:** 2025-06-27

**Authors:** Jinli Gao, Qinglang Wang, Jie Liu, Siqian Zheng, Jiahong Liu, Zhiyong Gao, Cheng Zhu

**Affiliations:** ^1^ Department of Psychiatry, the Affiliated Kangning Hospital of Wenzhou Medical University, Zhejiang Proving Clinical Research Center for Mental Disorder, Wenzhou, China; ^2^ Kangda College of Nanjing Medical University, Lianyungang, China; ^3^ Jiaxing Nanhu University School of Humanities and Arts Chinese Language and Literature N212, Jiaxing, China; ^4^ School of Mental Health, Zhejiang Provincial Clinical Research Center for Mental Disorders, The Affiliated Wenzhou Kangning Hospital, Wenzhou Medical University, Wenzhou, China

**Keywords:** depression, mouse model, artificial intelligence, gene co-expression network, random forest, neurobiology, cognitive dysfunction

## Abstract

**Introduction:**

An AI-assisted deep learning strategy was applied to analyze the neurobiological characteristics of depression in mouse models. Integration of weighted gene co-expression network analysis (WGCNA) with the random forest algorithm enabled the identification of critical genes strongly associated with depression onset, offering theoretical support and potential biomarkers for early diagnosis and precision treatment.

**Methods:**

Gene expression data from depression-related mouse models were obtained from public GEO datasets (e.g., GSE102556) and normalized using Z-score transformation. WGCNA was employed to construct gene co-expression networks and explore associations between modules and depression-like behavioral phenotypes. Depression-related gene modules were identified and subjected to feature selection using the random forest model. The biological relevance of selected genes was further assessed, and model accuracy was validated through performance evaluation.

**Results:**

Our findings revealed significant differential expression of genes such as Oprm1, BDNF, Tph2, and Zfp769 in the depression mouse model (p < 0.05). Notably, Oprm1 exhibited the highest feature importance, contributing to a model accuracy of 94.5%. Gene expression patterns showed strong consistency across the prefrontal cortex (PFC) and nucleus accumbens (NAC).

**Conclusion:**

The combined application of machine learning and transcriptomic analysis effectively identified core neurobiological genes in a depression model. Genes including Oprm1 and BDNF demonstrated functional relevance in modulating neural activity and behavior, offering promising candidates for early diagnosis and individualized treatment of depression.

## Introduction

Depression is one of the most prevalent mental health issues worldwide. Estimates from the World Health Organization (WHO) indicate that approximately 300 million individuals are affected worldwide (1, 2). Depression not only severely impacts patients’ quality of life but also increases the risk of suicide, imposing a significant burden on families and society (3, 4). Although advancements have been made in depression treatment, early diagnosis and effective therapeutic interventions remain substantial challenges (5, 6). Current diagnostic methods primarily rely on subjective assessments of clinical symptoms, which are susceptible to individual interpretation and may contribute to misdiagnosis or delayed detection (7). Moreover, treatment responses vary widely among patients, and existing therapeutic approaches are not universally effective. These challenges underscore the urgent need for precision medicine in the management of depression (8).

Advancements in genomics have prompted investigations into the genetic and molecular mechanisms underlying depression. Gene expression analysis, particularly genome-wide expression profiling, has opened new avenues for elucidating the biological basis of depression (9, 10). Large-scale bioinformatics datasets have facilitated the identification of numerous genes and biological pathways associated with depression (11). For example, the abnormal expression of genes such as brain-derived neurotrophic factor (BDNF) and serotonin transporter (SERT) has been closely linked to the pathogenesis of depression. Analytical approaches such as weighted gene co-expression network analysis (WGCNA) have been employed to identify critical genes and pathways involved in depression (12). These tools facilitate the investigation of complex gene interaction networks in the context of the disease (13).

Animal models, particularly mouse models, are invaluable tools for studying the neurobiological mechanisms of depression. By mimicking human behaviors and physiological responses, mouse models provide a platform to explore the underlying mechanisms of disease progression and evaluate potential therapeutic strategies (14). In depression research, stress-induced or genetically modified mouse models exhibit behavioral traits similar to those observed in human depression (15), such as behavioral inhibition, anxiety, and cognitive dysfunction. These models enable the investigation of neurobiological alterations associated with depression at the molecular level (16), including neurotransmitter system dysregulation and altered neuroplasticity, which are key factors in the pathophysiology of depression (17, 18).

An artificial intelligence (AI)–assisted machine learning approach was employed to analyze gene expression profiles derived from depression mouse models. The analytical framework integrated WGCNA with a random forest algorithm, enabling efficient processing of large-scale transcriptomic data and identification of key genes and biomarkers associated with depressive pathology (19, 20). WGCNA facilitated the detection of co-expression patterns across samples and the construction of gene modules, while the random forest model assessed the importance of each gene in classification tasks, supporting the selection of candidate genes with predictive value for disease differentiation (21). Application of this combined strategy enabled comprehensive interpretation of genome-wide transcriptional alterations in depression and enhanced the accuracy of disease prediction, thereby contributing to the development of individualized treatment strategies.

The primary objective of this study was to analyze gene expression data from a depression mouse model using advanced AI technologies and machine learning algorithms. This approach aimed to identify key genes and biomarkers closely associated with the onset and progression of depression. Identification of these biomarkers is expected to provide novel theoretical foundations and experimental evidence for early diagnosis and therapeutic development. From both scientific and clinical perspectives, the findings hold the potential to advance precision medicine in the context of depression, particularly in the design and implementation of personalized treatment strategies. By identifying and validating new disease biomarkers and therapeutic targets, this research aims to offer more effective and safer treatment options for patients with depression, ultimately optimizing depression management and significantly improving patient quality of life.

## Materials and methods

### Gene data collection in depression mouse models

A large number of analyzable gene expression datasets related to depression in mice were obtained from the publicly available GEO database (PMID: 28825715, GSE102556). To minimize the influence of uncontrollable confounding variables in the machine learning analysis, only mouse-derived data were included. The selected dataset comprised samples from the prefrontal cortex (PFC) and nucleus accumbens (NAC), including 9 male and 10 female CUS-treated mice and 10 male and 9 female control mice in the PFC group, as well as 10 male and 10 female CUS-treated mice and 10 male and 10 female control mice in the NAC group. Although both sexes were included, the analysis focused on gene expression changes in the PFC and NAC under chronic unpredictable stress (CUS), and sex was not included as an independent variable in the statistical models. In this dataset, gene expression changes were assessed in the NAC and PFC before and after exposure to CUS. These transcriptional alterations corresponded to variations in neurotransmitter regulation and behavioral performance, thereby exhibiting distinct neurobiological characteristics.

### Visualization and preprocessing of gene expression data in depression mouse models

**Figure 1:**
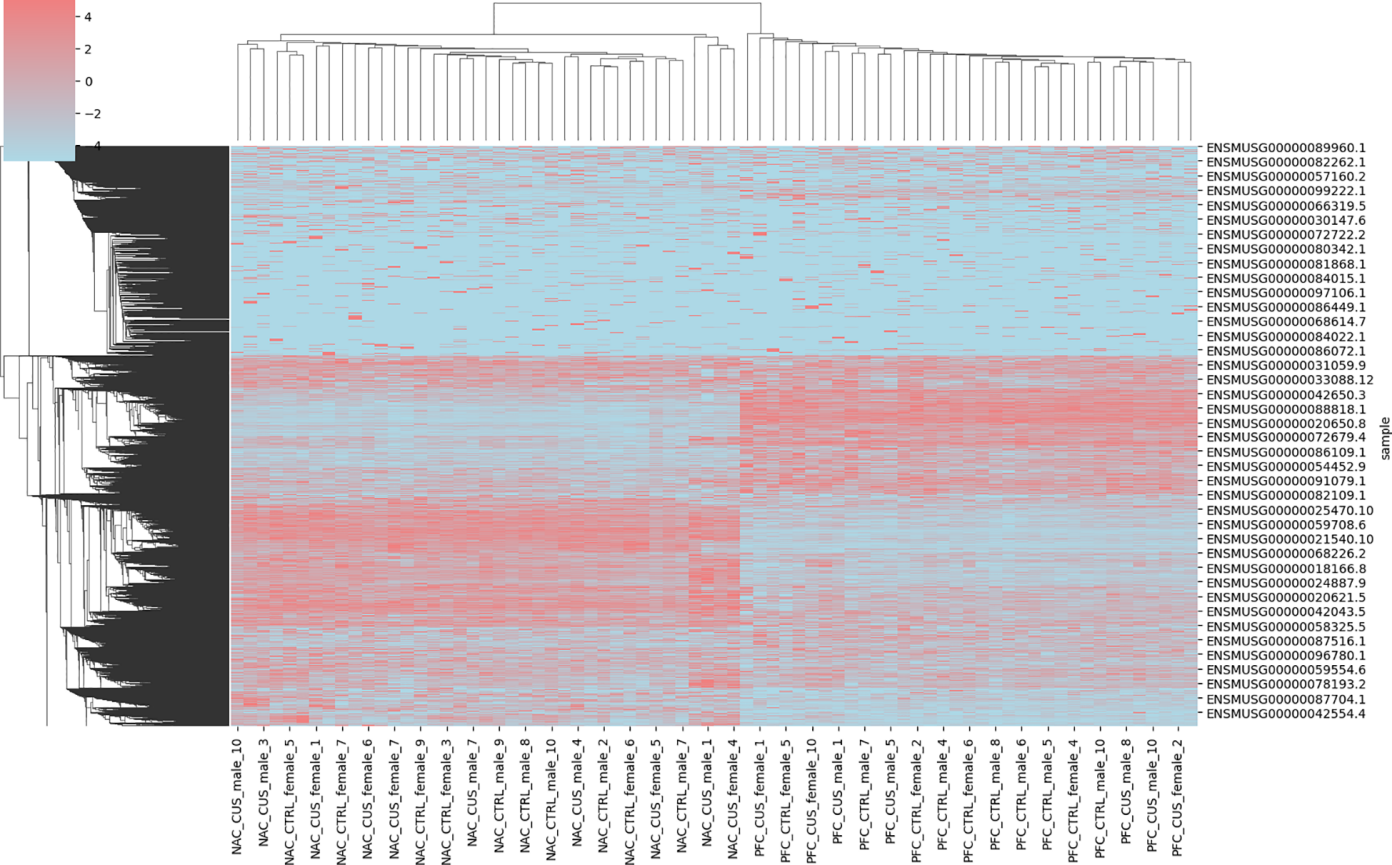
Visualization of the head and neck ultrasound dataset. Hierarchical clustering heatmap showing Z-score normalized expression values of differentially expressed genes across brain regions (NAC and PFC) and treatment conditions (CUS and control). Each row represents a gene (labeled by Ensembl ID), and each column corresponds to a sample. Samples are divided into four groups: NAC control (NAC_CTL), NAC CUS-treated (NAC_CUS), PFC control (PFC_CTL), and PFC CUS-treated (PFC_CUS), as indicated on the x-axis. Red and blue indicate upregulation and downregulation, respectively. The heatmap reveals distinct transcriptional signatures associated with both brain region and stress exposure.

Gene expression data from depression mouse models were analyzed following Z-score normalization, which standardized values within the range of –5 to 5. This normalization process reduced variability across genes and enabled more consistent and interpretable comparisons between experimental groups, as illustrated in **Figure 1**. Normalized data revealed trends in gene expression across distinct conditions.

The comparative analysis focused on two key brain regions: the NAC and the PFC. Significant differential gene expression was identified in both regions. The NAC, a brain region involved in reward processing and motivation, exhibited gene expression changes potentially associated with depressive symptoms such as anhedonia and diminished drive. In contrast, the PFC, which is involved in decision-making, emotional regulation, and social behavior, showed gene expression alterations that may correspond to cognitive and affective dysfunction commonly observed in individuals with depression.

Significant gene expression changes were detected across multiple brain regions following CUS testing. These changes were evident in the absolute values of gene expression and overall expression patterns. The observed transcriptional shifts suggest a substantial impact of CUS on the physiological and psychological states of the mice, subsequently influencing the expression of related genes. These findings provide crucial insights into the biological basis of depression and imply the involvement of region-specific mechanisms in different brain areas. Analysis of the dataset demonstrated its significant value for depression research. Standardized gene expression profiles established a reliable foundation for subsequent bioinformatics analyses, including accurate gene function annotation and pathway analysis. In addition, differential expression patterns in the NAC and PFC regions highlighted potential biomarkers that may contribute to the early diagnosis of depression and the development of targeted therapeutic strategies.

### Neurobiological feature identification model for depression in mice using WGCNA and random forest

**Figure 2:**
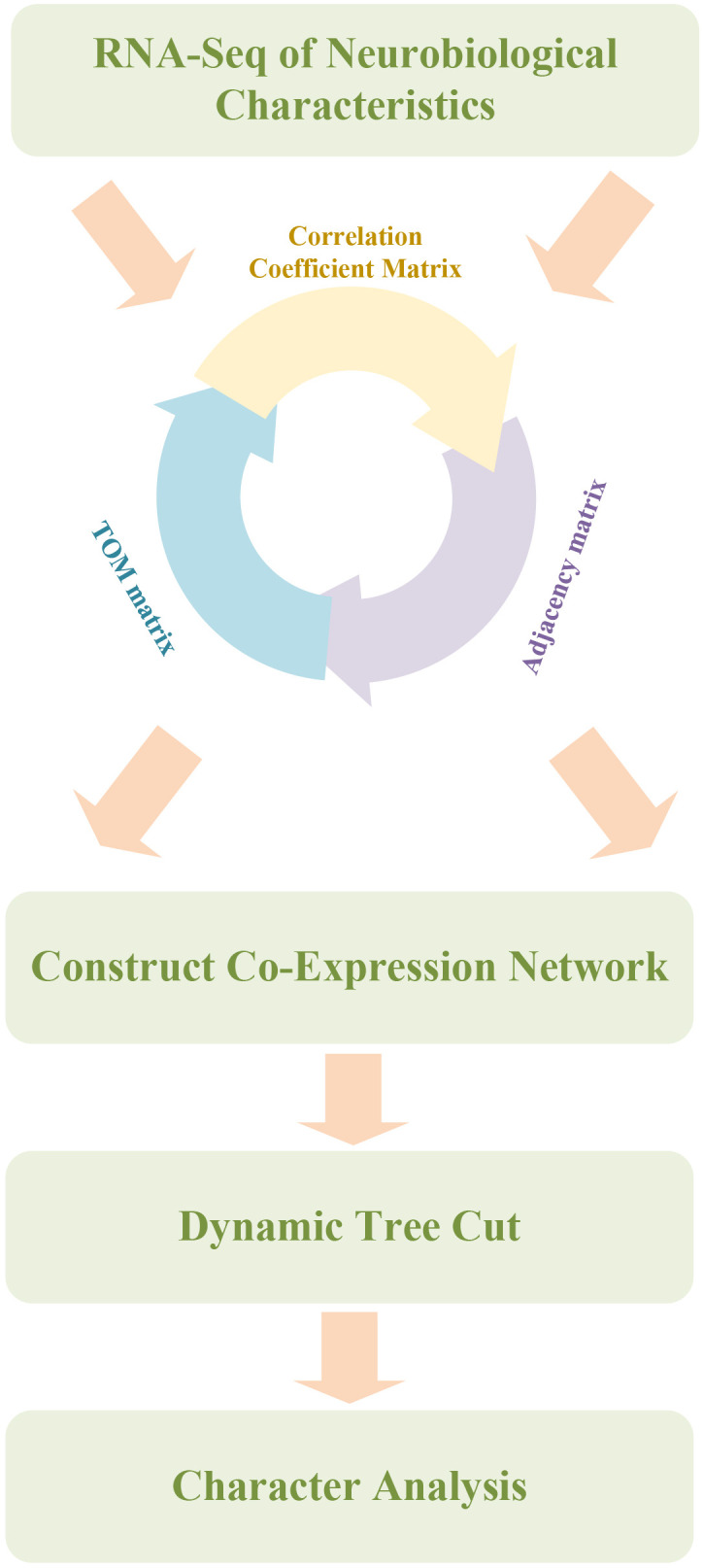
Depression mouse neurobiological feature identification model combining WGCNA and random forest. Workflow integrating Weighted Gene Co-Expression Network Analysis (WGCNA) with Random Forest for feature gene identification. The pipeline begins with the construction of a gene-gene correlation matrix based on RNA-seq data, followed by conversion into an adjacency matrix and a topological overlap matrix (TOM). Gene modules are identified using dynamic tree cutting. Subsequently, a Random Forest algorithm ranks genes based on their classification contribution, enabling selection of candidate neurobiological features that differentiate CUS and control mice.

To better understand the biological basis of depression, systems biology approaches have been increasingly employed, integrating genomics and bioinformatics tools to identify neurobiological features associated with depression. A neurobiological feature identification model was developed based on WGCNA and a random forest algorithm, as illustrated in **Figure 2**. The goal was to provide novel insights for the early diagnosis and personalized treatment of depression.

High-throughput gene expression profiling was first performed on brain tissue samples from depression mouse models. WGCNA was then used to construct a gene co-expression network and identify modules consisting of genes with similar expression patterns. The correlation between the module eigengenes and depression-related behavioral phenotypes was analyzed to select candidate modules potentially associated with the phenotype (22). Following module identification, relationships between selected modules and depression phenotypes were evaluated by calculating the correlations between module eigengenes and depression-related behaviors. Modules exhibiting strong associations were considered critical in the onset and progression of depression. This analytical step offered key insights into the molecular mechanisms underlying depressive states and established the foundation for subsequent feature selection.

Identified gene modules were subsequently integrated with a random forest model to calculate distinct neurobiological features. Random forest, an ensemble learning algorithm with strong classification and regression capabilities (23), was selected for its effectiveness in analyzing high-dimensional omics data characterized by a high feature-to-sample ratio and complex nonlinear relationships. As a bagging-based method, the model resists overfitting, making it especially suitable for gene expression datasets where the number of features far exceeds the number of samples. In addition, random forest does not impose strict assumptions on input variable distributions and can effectively capture nonlinear interactions, allowing for accurate modeling of multi-gene feature sets derived from WGCNA. The algorithm also generates interpretable feature importance scores, which aid in identifying potential key genes and support downstream biological interpretation. Compared to other black-box models such as support vector machines (SVMs) or neural networks, random forest offers greater interpretability, facilitating integration with functional annotation analyses. Therefore, the random forest model was chosen in this study for its balanced performance, stability, and biological interpretability, making it the optimal method for our research objectives.

The random forest algorithm was applied to model gene features extracted from WGCNA to identify neurobiological characteristics associated with depression. The dataset was divided into training and testing sets to evaluate model performance and to determine gene features significantly contributing to depression classification. Cross-validation was used during model training to optimize parameters and enhance predictive accuracy. The model generated classification outcomes and feature importance scores, which highlighted genes most relevant to depression-related neurobiological traits. These results established a basis for further biological validation and clinical application.

In summary, this study successfully developed a neurobiological feature identification model for depression in mice by combining WGCNA and the random forest model. The model revealed co-expression networks of depression-associated genes and identified key neurobiological features relevant to disease mechanisms. This research provides novel perspectives for the discovery of biomarkers and personalized treatments for depression. Further research may investigate the clinical relevance of the identified neurobiological features. Comprehensive biological validation is expected to generate evidence supporting early diagnosis and intervention, thereby advancing progress in depression research and mental health care.

### Statistical analysis

A combination of software tools and statistical methods was used to construct a neurobiological feature identification model for depression in mice. Data preprocessing and analysis were performed in Jupyter Notebook using Python. Libraries like pandas and Matplotlib were used for data loading, cleaning, and visualization. After obtaining the raw expression matrix from the GEO database, genes with extensive missing values were removed, and Z-score normalization was applied to constrain expression values within the range of –5 to 5. This transformation ensured comparability across samples and prepared the dataset for subsequent machine learning modeling. For statistical analysis, independent samples t-tests (Student’s t-test) were used to evaluate the significance of group differences, such as between CUS and control groups, based on normalized gene expression values. The combination of probability-based model output and hypothesis testing ensured statistical robustness and enhanced the biological interpretability of the results.

The Random Forest algorithm was implemented using the Scikit-learn library. Genes from WGCNA modules were selected as candidate features and used as input variables. The dataset was randomly divided into training (80%) and testing (20%) subsets to reduce overfitting and ensure generalizability. During model construction, 100 decision trees (n_estimators = 100) were used without constraints on depth (max_depth = None), and Gini impurity was adopted as the splitting criterion. Five-fold cross-validation was conducted to tune hyperparameters and assess stability. The optimal parameters were selected based on mean values of AUC and accuracy across folds. Feature importance was computed using the Gini importance index, indicating each gene’s relative contribution to model predictions. Final evaluation was performed on the test set, and metrics such as accuracy, precision, recall, specificity, and AUC were calculated to assess classification performance.

During data processing, Z-score normalization eliminated differences in feature scale, and incomplete data were excluded to maintain training consistency. Visualizations were created using Matplotlib and Visio to support data interpretation. Venn diagrams were used to display overlapping neurobiological features, and bubble plots illustrated functional patterns of candidate genes. Feature distribution and classification results were presented to reveal the model’s behavior under different conditions. Integration of statistical methods with visualization techniques enabled comprehensive validation of model performance. Analytical results supported the robustness and reliability of the modeling approach, offering a reference for future depression-related research and clinical application.

## Results

### Visualization of normalized gene expression data in depression mouse models

**Figure 3:**
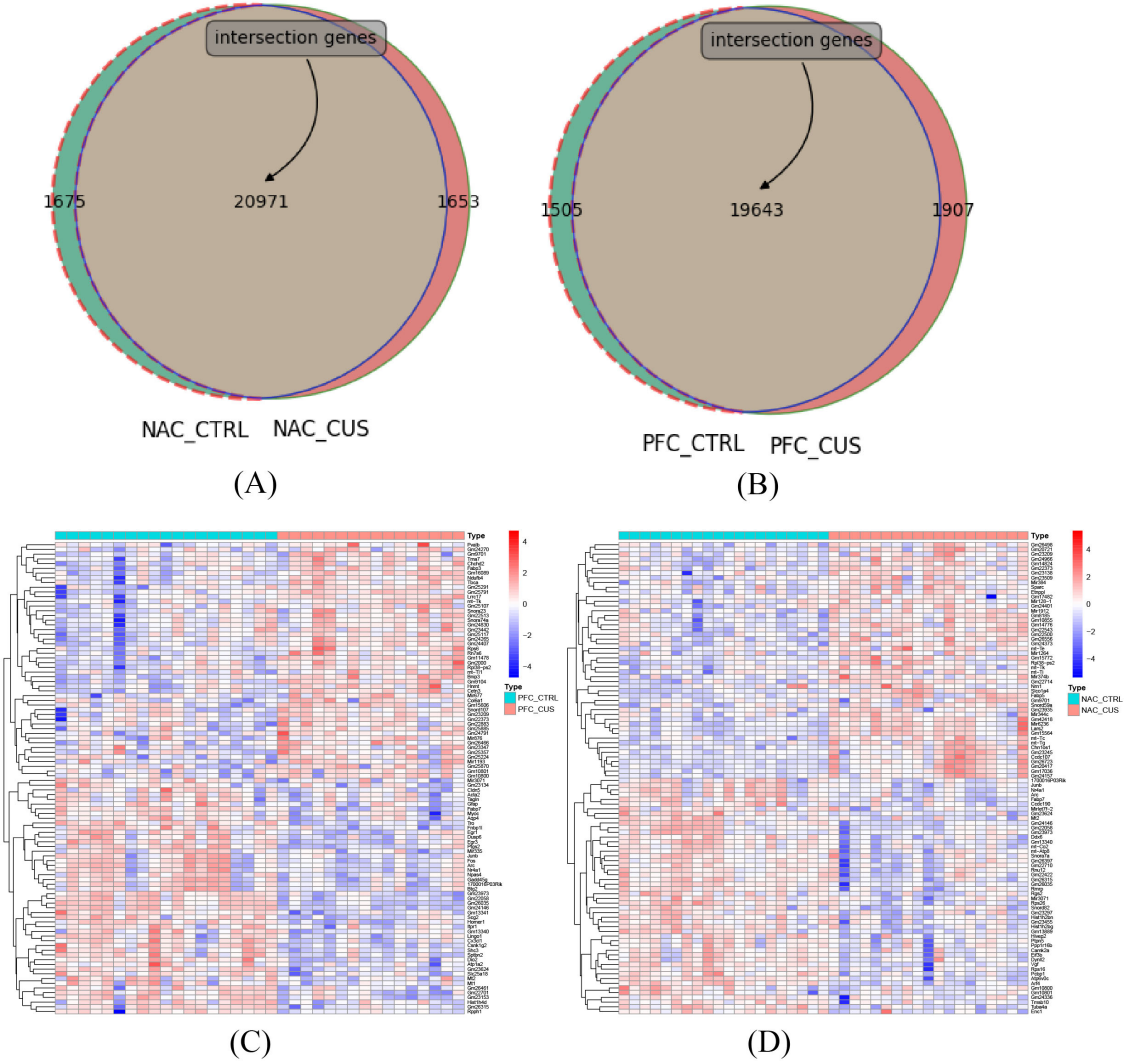
Venn diagram of gene expression counts across different brain regions. **(A)** Venn diagram comparing gene expression between the NAC control group (NAC_CTRL) and the CUS-treated group (NAC_CUS). A total of 20,971 genes were commonly expressed, with 1,675 and 1,653 genes uniquely expressed in each group, respectively, indicating notable transcriptomic changes in this region under CUS exposure. **(B)** Venn diagram comparing the PFC control group (PFC_CTRL) and the CUS-treated group (PFC_CUS). A total of 19,643 genes were shared between the two groups, with 1,505 and 1,907 genes uniquely expressed in each group, respectively. **(C)** Heatmap showing the top 50 differentially expressed genes between NAC_CTRL and NAC_CUS. **(D)** Heatmap showing the top 50 differentially expressed genes between PFC_CTRL and PFC_CUS.

Venn diagrams were employed to compare gene expression in two distinct brain regions, the NAC and the PFC, as shown in **Figure 3**. This analysis aimed to uncover the gene expression characteristics of specific brain regions in response to CUS in depression mouse models. According to the results of the Venn diagrams, 20,971 genes were commonly expressed between CUS-treated and control mice in the NAC region, while 19,643 genes were shared in the PFC region (**Figures 3A, B**), indicating extensive gene expression remodeling in both regions under chronic stress. To further characterize transcriptional alterations, the top 50 differentially expressed genes were visualized for comparisons between nucleus accumbens (NAC) control group (NAC_CTRL) and the chronic unpredictable stress-treated group (NAC_CUS), as well as between the prefrontal cortex (PFC) control group (PFC_CTRL) and the CUS-treated group (PFC_CUS) (**Figures 3C, D**). The top 50 differentially expressed genes showed distinct expression patterns between CUS-treated and control groups in both the NAC and PFC, indicating robust transcriptional responses to chronic stress. Despite functional differences between the two regions, both exhibited significant gene expression remodeling under stress conditions.

The NAC, a critical brain region associated with reward and motivation, displayed gene expression changes potentially linked to emotional regulation and behavioral responses in depression (24). A total of 20,971 co-expressed genes were identified, likely involving in neuroplasticity, inflammatory responses, and neurotransmitter metabolism. These gene expression changes likely reflect stress-induced adaptive mechanisms, suggesting its pivotal role in the pathogenesis of depression. The PFC, an essential region for regulating cognition and emotion, also exhibited notable gene expression changes (25). The 19,643 co-expressed genes may be associated with decision-making, emotional regulation, and social behavior. Gene expression remodeling in the PFC may contribute to cognitive impairments and emotional dysregulation, thereby aggravating depressive symptoms. These results highlight the critical role of the PFC in depression, particularly in emotion regulation and behavioral decision-making.

Comparison of gene expression between the NAC and PFC regions provided deeper insight into the neurobiological mechanisms underlying depression. The findings revealed region-specific transcriptional responses to stress and identified potential biomarkers with relevance to disease onset and progression. Co-expressed genes detected in both regions may serve as candidate targets for the early diagnosis and personalized treatment of depression, contributing to advances in mental health research. Future studies are warranted to investigate the specific functions of these genes in various brain regions and their role in the pathogenesis of depression, providing scientific evidence for developing novel therapeutic strategies.

### Visualization of WGCNA-based gene data analysis

**Figure 4:**
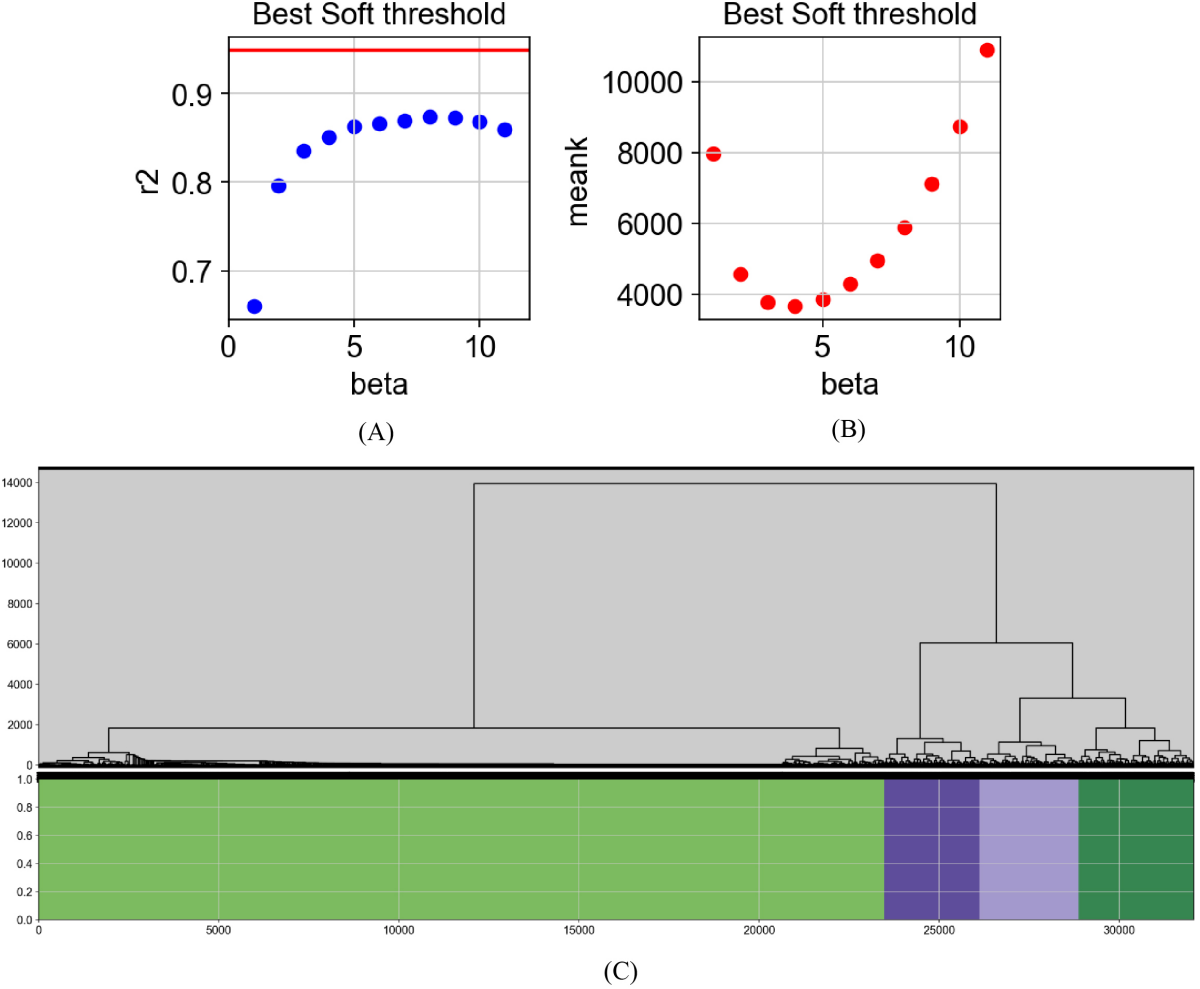
Visualization of gene analysis based on WGCNA. Network construction process using WGCNA. **(A)** Scale-free topology fit index (r²) plotted against soft-thresholding power (β); higher r² values indicate better fit to scale-free network criteria, typically exceeding 0.8. **(B)** Mean connectivity plotted against β values, showing a decreasing trend with increasing β. The optimal β is selected where r² first exceeds 0.8. **(C)** Gene modules identified via dynamic tree cutting on the TOM; modules consist of genes with similar expression patterns and likely shared biological functions.

To systematically identify key gene clusters involved in the regulation of depression, WGCNA was applied to construct modules and visualize co-expression networks based on transcriptomic data from depression mouse models. The goal was to identify gene clusters with highly correlated expression patterns and lay the foundation for subsequent phenotype correlation analysis (26, 27). Visualization of model training metrics enabled qualitative assessment of neurobiological characteristics, as shown in **Figure 4**.

**Figure 5:**
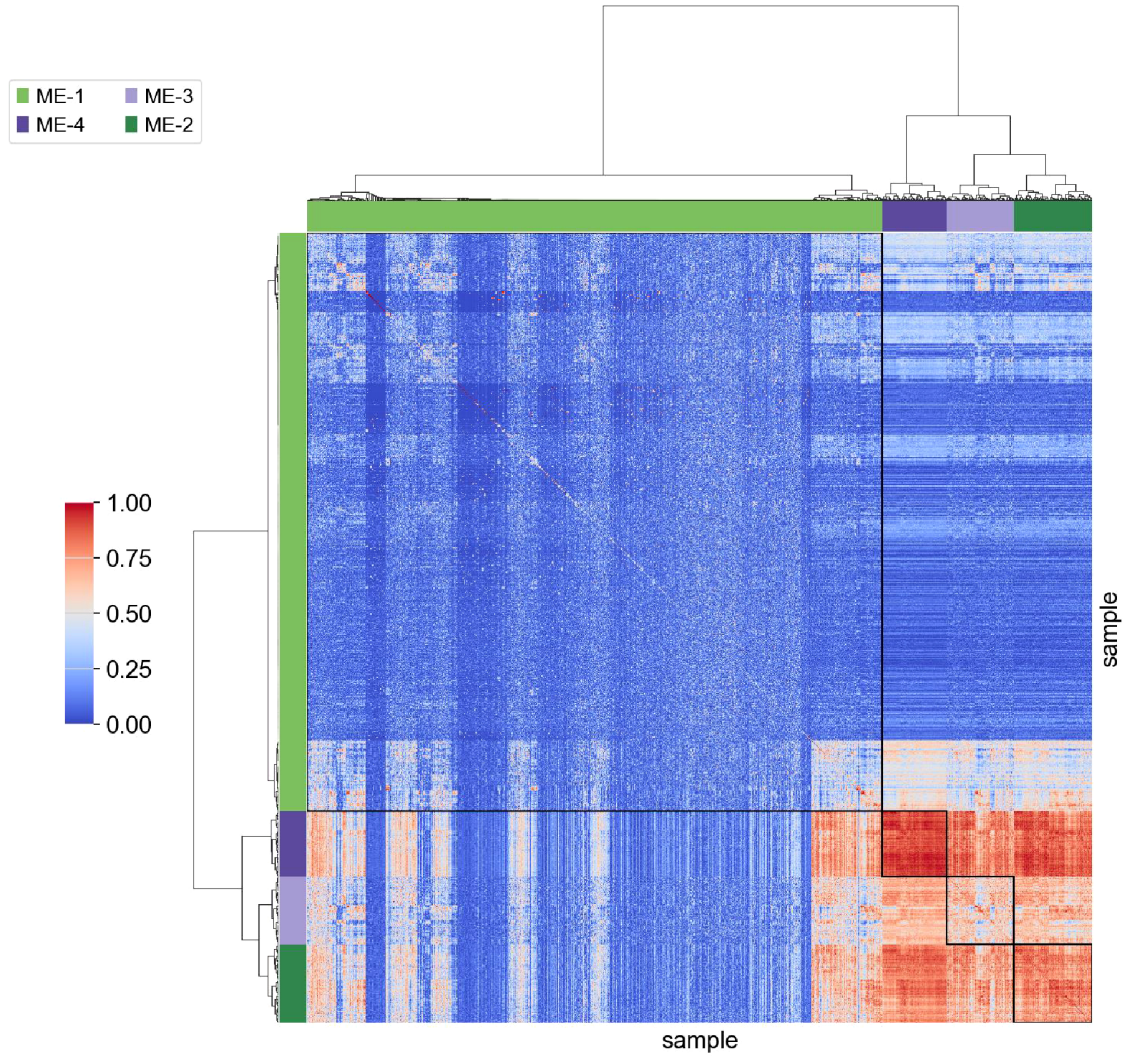
Visualization of the TOM between gene modules. TOM values range from 0 to 1, with higher values indicating greater similarity between gene modules. Smaller modules demonstrate higher internal similarity, suggesting potential functional or regulatory coherence. In contrast, larger modules exhibit lower overlap, indicating greater heterogeneity in gene function. Notably, Module 1 shows low similarity with other modules but contains the largest number of genes. The unique characteristics of this module suggest its potential relevance in depression-related pathways and warrant further investigation.

The topological overlap matrix (TOM) was visualized to examine the relationships among gene modules (**Figure 5**). TOM quantifies the similarity between gene pairs by considering both direct and shared connections, offering a more accurate representation of inter-gene relationships compared to simple correlation matrices. Hierarchical clustering of the TOM enabled the grouping of genes into modules with high topological overlap, often indicative of shared functional or regulatory roles. Modules identified through this process represent gene sets potentially involved in common biological pathways. Genes exhibiting high connectivity within each module were considered hub genes, likely to play central roles in network structure. Functional significance of these genes warrants further validation through differential expression analysis or phenotype correlation studies. In our study, the first gene module was identified as a key functional cluster. In summary, TOM visualization provided a comprehensive view of inter-module relationships and supported downstream steps such as module identification, functional annotation, and key gene discovery. These results contributed to a deeper understanding of gene function and interaction networks in the context of depression.

### Visualization of the results of the neurobiological characteristics recognition model for depression mice

**Figure 6:**
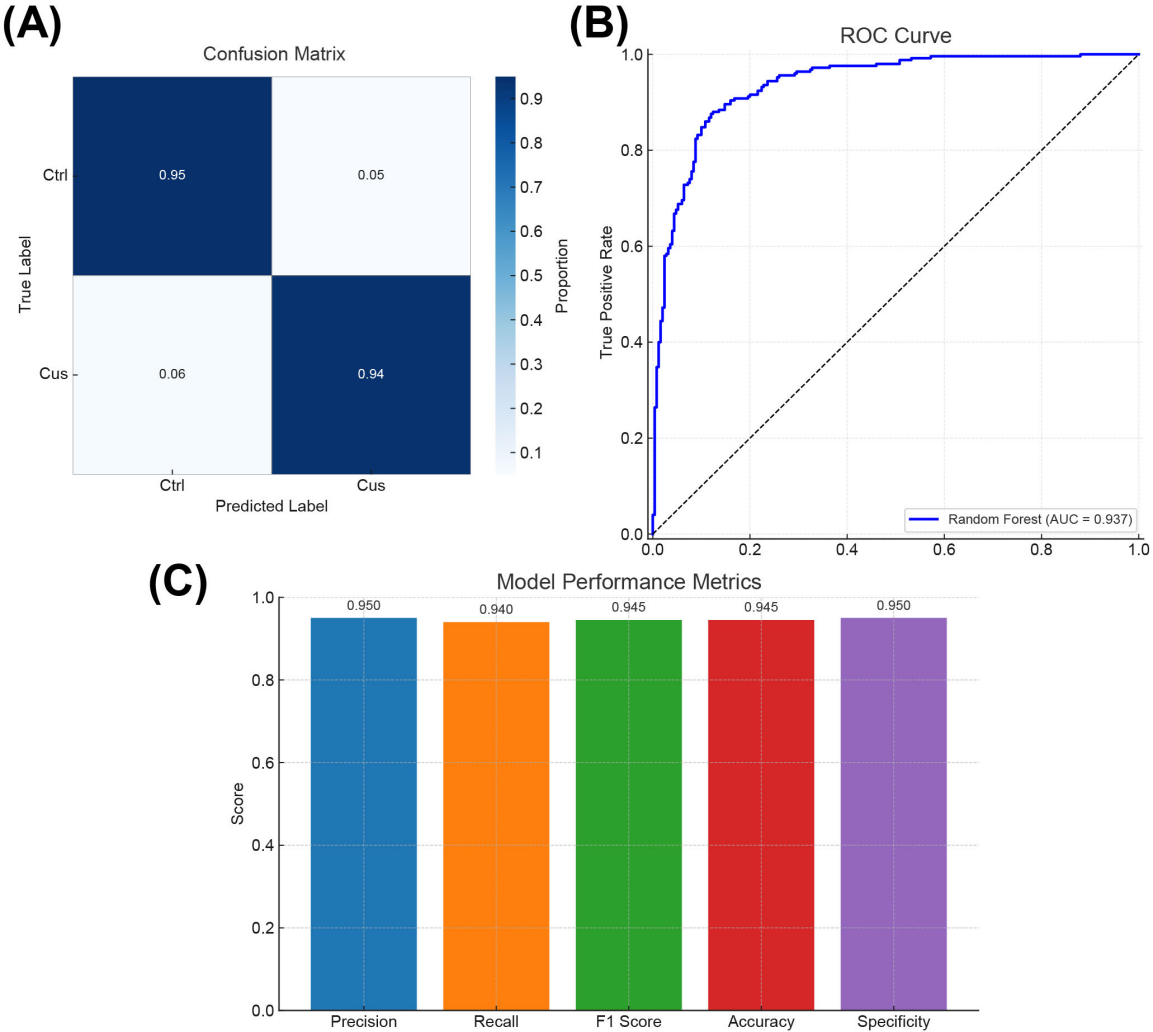
Visual performance evaluation of the random forest model. **(A)** Confusion matrix showing the classification accuracy of the model in identifying control (Ctrl) and chronic unpredictable stress (CUS) model mice. **(B)** ROC curve illustrating the model’s classification performance across various decision thresholds. **(C)** Bar chart presenting five classification metrics: Precision, Recall, F1 Score, Accuracy, and Specificity.

To systematically evaluate the performance of the constructed Random Forest model in identifying neurobiological features in depression mouse models, multi-dimensional visualizations were used, including a confusion matrix (**Figure 6A**), a receiver operating characteristic (ROC) curve (**Figure 6B**), and a bar chart summarizing multiple performance metrics (**Figure 6C**). These visual representations not only validated the model’s classification ability but also provided data support for subsequent optimization and feature interpretation.

The confusion matrix (**Figure 6A**) displayed the agreement between predicted and actual group labels. The results showed that the model achieved a classification accuracy of 95% for the control group (Ctrl) and 94% for the CUS model group, with an overall classification accuracy of 94.5%. These results demonstrated that the model accurately distinguished between the two neurobiological states. A few misclassifications were observed in the stress group, possibly reflecting intermediate or heterogeneous gene expression states, which may offer insights into classification boundaries and potential subtypes.

Model discrimination ability in probability space was further evaluated by plotting an ROC curve (**Figure 6B**). The area under the curve (AUC) reached 0.937, indicating strong predictive performance across varying classification thresholds. As a metric less sensitive to class imbalance, AUC provided a more robust evaluation of the model’s generalizability, supporting its applicability in diagnostic stratification and translational research.

A bar chart (**Figure 6C**) was generated to assess five key metrics: precision, recall, F1 score, accuracy, and specificity. Precision and specificity each reached 0.950, the recall was 0.940, and both F1 score and accuracy were 0.945 (**Supplementary Table S1**). These balanced results indicated consistent performance across positive and negative class predictions, a critical requirement in biomedical classification tasks to avoid prediction bias and improve model reliability.

In summary, the integrated evaluation using the confusion matrix, ROC analysis, and classification metrics confirmed the high accuracy, stability, and generalization ability of the Random Forest model in identifying neurobiological characteristics of depression in mice. The model effectively distinguished between control and CUS mice and provided a foundation for downstream identification of key genes and diagnostic marker systems. Future research may enhance the model’s diagnostic utility by integrating additional biological modalities (e.g., metabolomics, epigenomics) and behavioral data to further enhance its translational potential.

### Extraction and analysis of key genes in depression mouse model

**Figure 7:**
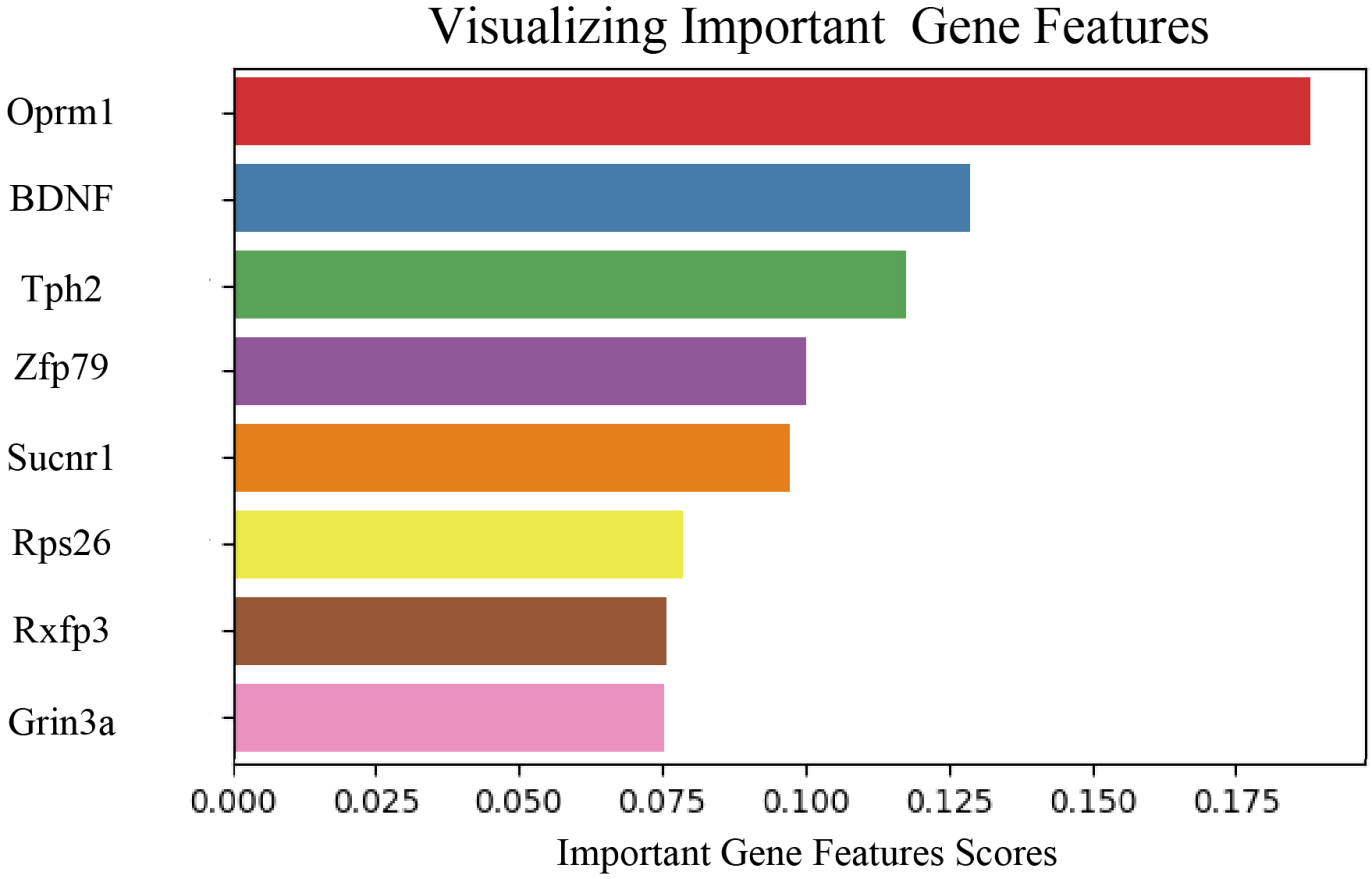
Visualization of feature extraction for each layer in the prognostic model. Bar plot displaying the relative contribution of eight genes calculated by the Random Forest model. The horizontal axis represents the importance score of each gene (Important Gene Features Scores), with higher values indicating greater weight in the model’s classification decisions.

To investigate gene expression changes associated with neurobiological characteristics of depression, eight key genes were identified through feature extraction, as shown in **Figure 7**. The selected genes included Oprm1, BDNF, tryptophan hydroxylase 2 (Tph2), Zfp769, Sucnr1, ribosomal protein S26 (Rps26), Rxfp3, and Grin3a. Further analysis of these eight genes (**Table 1**) in the depression mouse model revealed the following insights:

1. Oprm1: This gene encodes the μ-opioid receptor involved in mood and pain regulation (28). Altered expression in depression models has been linked to emotional dysregulation (29).
2. BDNF: BDNF plays a key role in neuroplasticity, and its downregulation has been associated with depressive symptoms and cognitive dysfunction (30).
3. Tph2: Tph2, a rate-limiting enzyme in serotonin synthesis, shows reduced expression in depressive states, implicating serotonergic dysfunction (31).
4. Zfp769: This gene encodes a zinc finger protein involved in gene regulation. Members of this family have been associated with neurodegeneration and chronic neuroinflammation (32).
5. Sucnr1: Sucnr1, a receptor for short-chain fatty acids, participates in metabolic signaling and exhibits differential expression in models of central nervous system inflammation (33).
6. Rps26: Rps26, a component of the ribosomal machinery, has been implicated in altered protein synthesis in neuropsychiatric disorders, including anorexia nervosa (34).
7. Rxfp3: This gene encodes a receptor involved in regulating neuroendocrine functions such as stress response, arousal, feeding, and cognition. Its relevance to neurological conditions has been widely reported (35).
8. Grin3a: This gene is related to neuroendocrine function. Altered expression has been observed in depressive mouse models, suggesting a role in emotional regulation (36).

**Table 1:** Key genes identified in the depression mouse model and their functions with supporting literature.

Gene	Full name	Functional analysis and literature support
Oprm1	opioid receptor mu 1	Encodes the μ-opioid receptor, involved in pain and emotion regulation (28); expression changes observed in depression mouse models, possibly affecting emotional response (29).
BDNF	brain derived neurotrophic factor	Plays a key role in neuroplasticity; low levels are associated with depression, affecting learning and memory (30).
Tph2	tryptophan hydroxylase 2	A key enzyme in serotonin synthesis; decreased expression is closely associated with the onset of depressive symptoms (31).
Zfp769	zinc finger protein 769	Encodes a zinc finger protein involved in gene regulation; associated with susceptibility to neurodegeneration and chronic inflammation (32).
Sucnr1	succinate receptor 1	Encodes a short-chain fatty acid receptor involved in metabolic regulation; differentially expressed in chronic CNS inflammation (33).
Rps26	ribosomal protein S26	Involved in protein synthesis; plays a role in the neurobiology of anorexia nervosa, indicating disrupted subcortical appetite circuits (34).
Rxfp3	relaxin family peptide receptor 3	Encodes a receptor that regulates neuroendocrine functions; involved in feeding, stress, arousal, and cognition; potential target for neurological disorders (35).
Grin3a	—	Related to neuroendocrine function; expression changes observed in depressed mice, potentially affecting stress response and emotional states (36).

Collectively, these genes contribute to diverse regulatory pathways, including neurotransmitter metabolism (Tph2), neurotrophic signaling (BDNF), metabolic regulation (Sucnr1), and ribosomal function (Rps26), supporting their multilayered regulatory roles in the development of depression.

**Figure 8:**
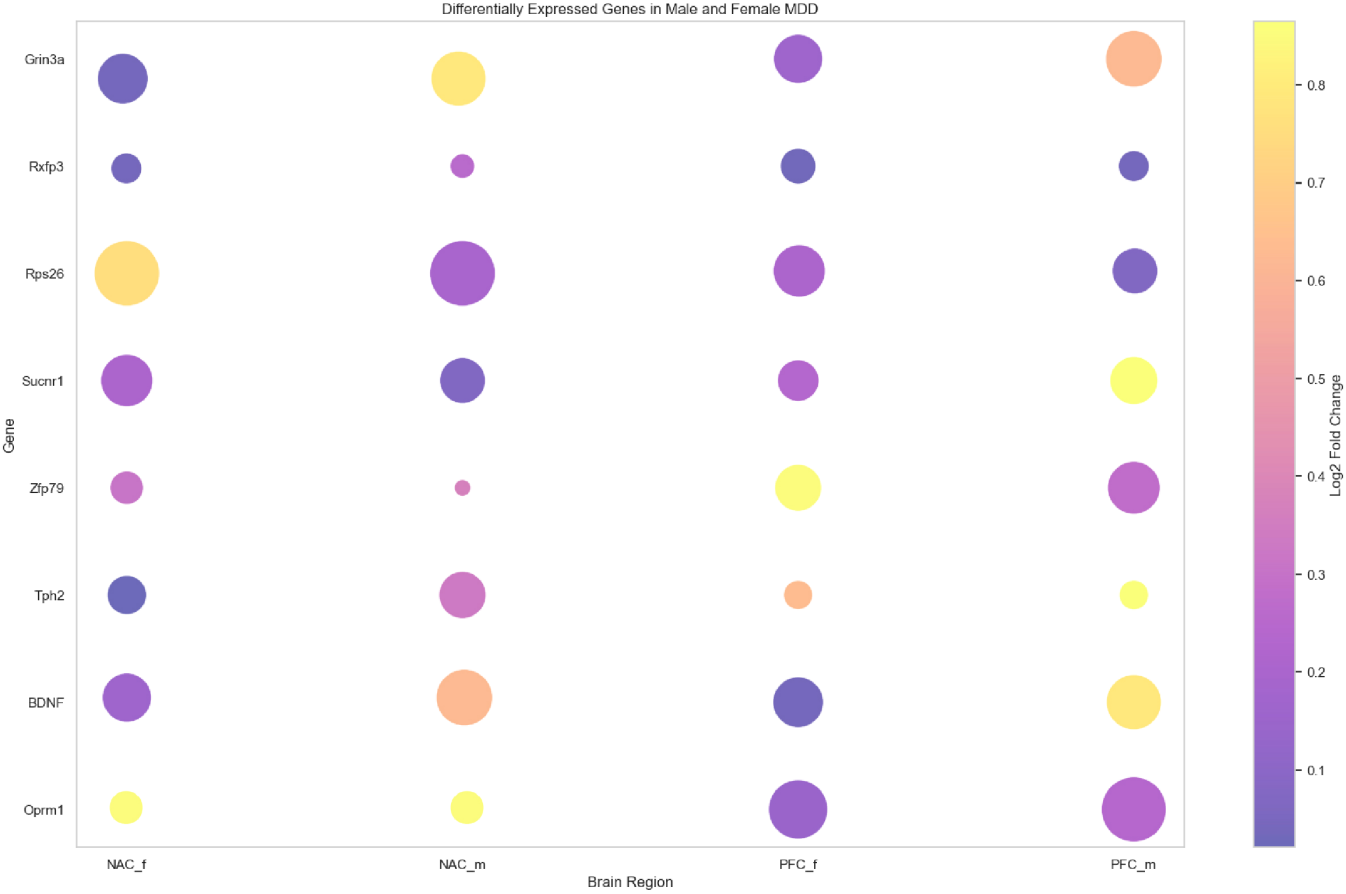
Visualization of the bubble plot for eight key genes. Bubble plot representing expression levels and statistical significance of eight key genes (Oprm1, BDNF, Tph2, Zfp769, Sucnr1, Rps26, Rxfp3, and Grin3a) across different brain regions—nucleus accumbens (NAC) and prefrontal cortex (PFC)—and sexes (male: _m, female: _f). Bubble size reflects significance (inverse of p-value), and color intensity indicates Log_2_ fold change in expression. The color scale is shown on the right.

To further investigate the role of these eight genes in the neurobiological characteristics of depression in mouse models, gene expression data were visualized using bubble plots across different brain regions (**Figure 8**). In the plot, circle size represents the significance level of gene expression in each region, while the color reflects the expression level. This approach enabled a comparative analysis of spatial expression patterns and their associations with depressive phenotypes. Among the eight genes, Oprm1, BDNF, Tph2, Zfp769, Sucnr1, Rps26, and Grin3a showed high significance levels across multiple brain regions, while Rxfp3 displayed relatively lower significance (**Supplementary Table S2**). These findings suggest that most of the identified genes may play essential roles in the molecular mechanisms underlying depression in mice. For instance, the high significance level of the Oprm1 gene supports its involvement in mood regulation and nociceptive processing (29). Elevated expression and significance of BDNF further confirm its established role in neuroplasticity and emotional regulation (30). The high expression of Tph2, related to serotonin synthesis, may contribute to the modulation of mood and emotional responses (31).

Furthermore, the significance levels of Zfp769, Sucnr1, Rps26, and Grin3a suggest potential regulatory functions in the stress response and depressive phenotypes. Altered expression patterns of these genes may reflect adaptive neurobiological responses to chronic stress, highlighting their relevance to the onset and progression of depressive disorders.

In summary, bubble plot visualization offered a clearer perspective on the functional relevance and potential mechanisms of the identified genes in the depression mouse model. These findings offered significant insights for further exploration of biomarkers and therapeutic targets for depression. Future studies may further explore the functional roles and molecular interactions of these genes to advance the understanding of depression pathogenesis.

## Discussion

Depression is one of the most burdensome neuropsychiatric disorders globally, with high incidence and recurrence rates emphasizing the urgent need for further research (3, 37, 38). Current diagnostic approaches primarily rely on the subjective assessment of clinical symptoms (39) and lack reliable objective biomarkers (40), posing significant challenges for early disease detection and personalized interventions (41). The present study applied AI techniques in combination with transcriptomic data to investigate neurobiological features in mouse models of depression. By identifying key regulatory genes and expression patterns, the analysis provided both theoretical insights and practical foundations for advancing objective diagnosis and personalized intervention in depression.

Theoretically, this project contributes to a deeper understanding of the pathogenesis of depression. The NAC and PFC are critical brain regions that regulate mood and cognitive functions and are closely associated with the pathophysiology of depression (38). However, existing studies are mainly limited to single-level analyses and fail to uncover the complex gene regulatory networks between brain regions (42). In this study, WGCNA was used to identify gene modules with similar expression patterns, followed by correlation analysis between module eigengenes and depression-related behavioral phenotypes. Subsequently, a Random Forest model was employed for feature selection to identify candidate genes with high classification importance. This integrative approach revealed potential regulatory networks underlying depression. The multidimensional integrative analysis not only identified key functional genes and their interactions but also enabled the construction of molecular regulatory frameworks associated with depression. These findings advanced the understanding of the complex biological mechanisms underlying the disorder.

Furthermore, this study provides new insights into diagnosing and treating depression from a practical perspective. Analysis of transcriptomic data from depression-related mouse models in the GEO database enabled the identification of key genes, including Oprm1, BDNF, and Tph2, which exhibited marked expression differences between the NAC and PFC regions. These genes showed potential as biomarkers and may serve as candidate indicators for early diagnosis and clinical subtyping of depression. Additionally, machine learning models were employed to predict the functional importance of these genes, laying the groundwork for the development of targeted intervention strategies. The integration of transcriptomic analysis with artificial intelligence established a robust framework that supports the advancement of precision medicine approaches in depression research and clinical translation.

Finally, this study presents a significant methodological innovation by integrating WGCNA with the random forest algorithm. Traditional gene expression data analysis methods are often based on linear assumptions and fail to address complex non-linear relationships between genes. In contrast, the combined use of WGCNA and machine learning enabled the extraction of latent structure from high-dimensional data, effectively addressing limitations in handling biological complexity and variability. Furthermore, this study highlights the application of AI-assisted technologies in biomedical data analysis, providing a scalable research paradigm for investigating the molecular mechanisms and potential biomarkers of other neuropsychiatric disorders, such as anxiety and bipolar disorder.

The clinical relevance of the study lies in applying AI-assisted machine learning methods, such as WGCNA and the random forest model, to identify key genes associated with depression in a mouse model. Genes like Oprm1 and BDNF, which exhibited significant differential expression and strong functional relevance in the neurobiology of depression, emerged as potential molecular indicators. The analysis deepened understanding of the biological basis of depression while offering candidate biomarkers for early diagnosis and disease monitoring. Furthermore, these findings contribute to developing therapeutic strategies targeting specific molecular pathways, which may improve the personalization of depression treatment, offering more precise therapeutic options, potentially reducing side effects, and enhancing treatment efficacy.

Methodologically, the integration of machine learning techniques with nonlinear feature learning and systems biology-based network analysis demonstrated strong advantages in analyzing high-dimensional omics data. This combined strategy established a novel framework for investigating mechanisms of complex disease and emphasized the broader applicability of AI-assisted analysis in neuropsychiatric research.

However, several limitations should be acknowledged, particularly regarding the translational challenges of extending findings from mouse models to human research. Interspecies differences remain a critical barrier, given the substantial variation in brain anatomy, neural circuitry, developmental trajectories, and gene regulatory mechanisms between mice and humans. As a result, the functional relevance of genes such as Oprm1 and BDNF identified in mouse models warrants further validation in primate systems. Although certain brain regions involved in emotional regulation are evolutionarily conserved between rodents and humans, notable differences in synaptic transmission and hormonal response may limit the direct extrapolation of results.

Second, the differentially expressed genes identified in this study were analyzed and evaluated exclusively in mouse models, lacking validation in human samples. Future research should incorporate cross-validation strategies using publicly available human datasets (e.g., GTEx, PsychENCODE), peripheral blood transcriptomic data, and brain imaging-transcriptome datasets (e.g., fMRI-coupled RNA-seq) to ensure the expression consistency and stability of these genes in clinical samples.

To overcome the limitations of cross-species translation, the application of human induced pluripotent stem cells (iPSCs) offers a promising avenue. iPSCs, derived from skin or blood cells of patients with depression, can be differentiated *in vitro* into neurons, astrocytes, or three-dimensional brain organoids to more accurately simulate the biological state of the human nervous system. Thus, future research should prioritize three directions: (1) constructing humanized models (e.g., iPSC-derived neurons and brain organoids) to mimic human neurodevelopment and gene regulation; (2) integrating multi-omics data from large-scale clinical cohorts to conduct cross-validation and enhance external validity; and (3) promoting interdisciplinary collaboration with clinical psychiatry departments to conduct multi-center and real-world studies, thereby accelerating the translation of basic research into clinical applications.

## Conclusion

The present study systematically analyzed gene expression features in the NAC and PFC regions of depression mouse models using WGCNA and the Random Forest model. The results revealed 20,971 and 19,643 co-expressed genes in the NAC and PFC regions, respectively, in the CUS model, indicating extensive transcriptomic remodeling. The Random Forest model achieved an accuracy of 94.5% and an AUC of 0.937, demonstrating strong classification performance. Based on feature importance scores, eight key genes were identified, including Oprm1, BDNF, Tph2, Zfp769, with Oprm1 showing the highest contribution. Bubble plot visualization confirmed significant expression of these genes in critical brain regions, supporting their potential as biomarkers.

Collectively, these findings contribute to mechanistic insights into depression, demonstrate the feasibility of AI-assisted biomarker discovery, and provide a data-driven framework for diagnostic model development. Despite the inherent limitations of translating results from animal models to clinical settings, the study offers a theoretical basis and methodological roadmap for future research. Continued validation and refinement of these findings may facilitate the development of objective diagnostic tools and individualized treatment strategies, advancing precision medicine approaches in depression and yielding broader benefits for clinical practice and public mental health.

## Data Availability

The raw data supporting the conclusions of this article will be made available by the authors, without undue reservation.
