# Supplementary material for: Integrating weighted gene co-expression network analysis and machine learning to elucidate neural characteristics in a mouse model of depression
Source: Front Psychiatry. 2025 Jun 27;16:1564095. doi: 10.3389/fpsyt.2025.1564095 (PMC12245827; doi:10.3389/fpsyt.2025.1564095)
Supplement: Supplementary file 1 [file Table1.docx]

**Table S1. Summary of Classification Metrics for the Random Forest Model in Depression Mouse Classification.**

| Metric | Value |
| --- | --- |
| Accuracy | 0.945 |
| Precision | 0.95 |
| Recall | 0.94 |
| F1 Score | 0.945 |
| Specificity | 0.95 |
| AUC | 0.937 |

**Table S2. Statistical Analysis of Differential Expression of Eight Key Genes Across Brain Regions and Sexes.**

| Gene | Region | log₂FC | p-value |
| --- | --- | --- | --- |
| Oprm1 | NAC | 1.25 | 0.0004 |
| BDNF | PFC | -1.1 | 0.0012 |
| Tph2 | PFC | -0.95 | 0.0045 |
| Zfp769 | NAC | 0.88 | 0.0023 |
| Sucnr1 | NAC | -1.15 | 0.0009 |
| Rps26 | PFC | 0.73 | 0.0056 |
| Rxfp3 | PFC | 0.5 | 0.0921 |
| Grin3a | NAC | -1.02 | 0.0019 |
